# Tanshinone IIA and Astragaloside IV Inhibit miR-223/JAK2/STAT1 Signalling Pathway to Alleviate Lipopolysaccharide-Induced Damage in Nucleus Pulposus Cells

**DOI:** 10.1155/2021/6554480

**Published:** 2021-10-12

**Authors:** Xiaoxun Du, Xiaoying Wang, Kaiying Cui, Yungang Chen, Chao Zhang, Kang Yao, Yanke Hao, Yuanzhen Chen

**Affiliations:** ^1^Shandong University of Traditional Chinese Medicine, Jinan, Shandong 250014, China; ^2^Neck-Shoulder and Lumbocrural Pain Hospital of Shandong First Medical University, Shandong First Medical University & Shandong Academy of Medical Sciences, Jinan, Shandong 250014, China; ^3^Jinan Vocational College of Nursing, Jinan, Shandong 250014, China; ^4^Affiliated Hospital of Shandong University of Traditional Chinese Medicine, Jinan, Shandong 250014, China; ^5^Guangzhou University of Chinese Medicine, Guangzhou, Guangdong 510000, China

## Abstract

Astragaloside IV (AS IV) and tanshinone (TS IIA) are the main natural components of Salvia miltiorrhiza and Radix Astragali, respectively. The amalgam of TS IIA and AS IV has potential therapeutic value in many inflammation-related diseases. However, the aftereffect of TS IIA and AS IV for lumbar disc herniation is not clear. Although the function of miR-223 in the inflammation-related JAK/STAT pathway is unknown, it is particularly expressed in human degenerative nucleus pulposus cells. This study has investigated the efficacy of the combined application of TS IIA and AS IV in the treatment of intervertebral disc nucleus pulposus cells (NP cells) injured by lipopolysaccharide (LPS). After miR-223 inhibitor imitated NP cells, the state of the JAK family and STAT family was recognized by Western blotting (Western blot, WB) and reverse transcriptase quantitative polymerase chain reaction (qPCR). The shRNA lentivirus interference vector targeting the STAT family was constructed, and the NP cell line stably interfering with the STAT gene was established after transfection. The expression of TNF-*α*, IL-6, MMP-9, MMP-3, caspase-1, and caspase-3 was detected by lipopolysaccharide (WTNP cells), control virus NP cells, STAT downregulation NP cells, enzyme-linked immunosorbent assay (ELISA), Western blot, and qPCR, respectively. The cell survival rate was detected by flow cytometry and TUNEL staining reverse transcriptase-polymerase chain reaction (qPCR). NP cells were treated with TS IIA and AS IV which had been made into different concentrations, and then, the expression of miR-223, p-STAT1, and p-JAK families was detected by WB Western blotting and qPCR. MiR-223 selectively acts on JAK2/STAT1 pathway, increases the expression of TNF-*α*, IL-6, MMP-9, MMP-3, caspase3-1, and caspase-3, and induces apoptosis, which can be eliminated by silencing STAT1. TS IIA combined with AS IV could inhibit the expression of miR-223, p-STAT1, and p-JAK2 in NP cells, and they showed a dose-dependent tendency to p-STAT1 and p-JAK2. This study shows that miR-223 promotes the inflammatory response and induces cell injury of NP cells by acting on the JAK2/STAT1 pathway, and the combination of TS IIA and AS IV may protect NP cells by downregulating miR-223 and inhibiting the expression of JAK2 and STAT1.

## 1. Introduction

Lumbar disc herniation is a frequently occurring and common disease in orthopaedics, and the specific mechanism of lumbar intervertebral disc degeneration (IDD) is not very clear. With the development of biopharmaceuticals, some targeted antibodies and small molecular compounds have been found to have the effect of preventing and reversing IDD in animal experiments. Although the application prospect is not clear, it opens up a new idea for the treatment of lumbar disc herniation [1–3].

At present, the models needed to study IDD mainly include the mechanical induction model and damage induction model. These methods directly damage the intervertebral disc and destroy its immune immunity state, which is different from the process of human natural degeneration [4, 5]. Nucleus pulposus cells (NP cells) are the most important cells in the intervertebral disc [6, 7]. Because of their important role in maintaining the stability of the intervertebral disc, nucleus pulposus cells have become important cells in the field of tissue engineering for cell transplantation to repair degenerative intervertebral disc. It has been reported that inflammation and apoptosis in NP cells and extracellular matrix (ECM) are closely related to lumbar disc degeneration [8], mainly manifested by the activation of various matrix-degrading enzymes induced by inflammatory mediators, leading to ECM degradation, and these lesions are essential in degeneration of intervertebral disc. In inflammatory response, most cytokines can activate the JAK-STAT pathway through their receptors and activate the JAK signal to phosphorylate downstream STAT and bind the SH2 domain, thus producing downstream biological effects. Therefore, reducing inflammatory reactions and blocking the JAK-STAT pathway are of great significance in the treatment of IDD.

As a short noncoding RNA, miRNA was officially regarded as one of the classic gene regulators in eukaryotic cells in 2001 [9]. It plays an essential role in cell proliferation, genesis, development, and metabolism by acting on other genes [10]. It has been confirmed that miRNA is essential for the occurrence and development of IDD. For example, the overexpression of miRNA-21 targets phosphatase and tensin homologues (PTEN) directly, so that the PI3K/Akt pathway can be activated [11]. At present, the research on miR-223 is mainly focused on hematopoietic cells and immune granulocytes. MiR-223 can target transcription factor Mef2c, in miR-223 knockout mice to eliminate the expression of Mef2c and inhibit the expansion of myeloid progenitor cells [12, 13]. MiR-223 is considered by more and more people as a potential biomarker for the diagnosis and medicaments of inflammatory diseases for the higher level of miR-223 in the primitive stage of osteoarthritis than that in the latter part [14, 15]. Nevertheless, the exact role of miR-223 and its relationship with the JAK-STAT pathway is not clear.

Radix Salviae Miltiorrhizae and Radix Astragali are two kinds of drugs often used by traditional Chinese medicine doctors in the clinic. Tanshinone II A (TS IIA), as the foremost fat-soluble active component of Salvia miltiorrhiza, is often used in the prophylaxis and medication of cardiovascular diseases [16, 17]. TS IIA is an effective free radical scavenger that can inhibit the expression of chemokine [18–20]. However, the mechanism of these two anti-inflammatory compounds in NP cells is unknown. This study will explore how the JAK-STAT pathway of NP cells is regulated and ultimately affect apoptosis under the mediation of miR-223 and inflammatory environment, and study the effect of TS IIA combined with AS IV on the expression of miR-223 and the intervention on nucleus pulposus cells.

## 2. Method

### 2.1. Cell Culture

NP cells were cultured to passages 2-3 by DMEM complete medium that had been added with 10% fetal bovine serum (FBS) (121000061, Gibco, Shanghai, CHN), 100 U/ml penicillin (Sigma-Aldrich Chemical Company, St-Louis MO, USA), and 100 mg/ml streptomycin (Sigma-Aldrich Chemical Company, St-Louis MO, USA). TS IIA (B20257) and AS IV (B20564) which purity is greater than 98% were purchased from Shanghai Yuanye Bio-Technology Co. To make the concentration of DMSO 0.1%, TS IIA and AS IV were deliquesced in dimethyl sulfoxide (DMSO; Sigma-Aldrich). After that, they were intermixed with the culture medium. Cells of experimental group were given a variety of doses of TS IIA and AS IV. The negative control (NC) cells were treated with 0.1% DMSO.

### 2.2. Cell Transfection

To explore the effectiveness of miR-223 on NP cells, cell's endogenous expression was controlled. We obtained miRNA control, miR-223 inhibitor, and miR-223 mimics from Promega (Promega, MA, USA). The short hairpin RNA (shRNA) lentivirus interference vector was constructed for stat. The NP cell line which can stably interfere with the stat gene was established. We use LPS (10 ugs/ml) to induce NP cells with various lengths of time.

### 2.3. Quantitative Real-Time PCR (qRT-PCR) Analysis

We apply TRIzol reagent (Invitrogen) in concordance with supplier's protocol to isolate total RNA from cells. Subsequently, the first strand of transcripts was used to synthesize cDNA kits (Roche, Mannheim, Germany). After that, the reverse transcription was achieved. qRT-PCR was realized with SYBR Green I Master (Rox) on the LightCycler® 480 System (Rox). The U6 or GAPDH was identified as a standard about the relative gene. Their expression quantity was figured out applying the 2^−ΔΔCt^ method.

### 2.4. ELISA Determination

Expression levels of TNF-*α*, IL-6, MMP-3, and MMP-9 were measured by the use of the ELISA reagent kit (R&D Systems, Minneapolis, MN, USA). The research was put into practice accurately and exactly in conformity with the kit instructions.

### 2.5. Western Blot (WB)

To extract total protein, the NP cells which were treated by previous steps were cleaned with cold PBS 2–3 times. Then, they lysed with RIPA lysis buffer (Sigma-Aldrich, St. Louis, MO, USA). The concentration of the total protein was determined by the use of a BCA kit (Thermo Fisher Scientific, San Jose, CA, US). The primary antibodies were as follows: anti-p-JAK1, anti-p-JAK2, anti-p-JAK3, anti-caspase-1, anti-caspase-3, anti-p-STAT1, and anti-GAPDH. Afterwards, the transfer membrane was cleaned by TBST 3 times. Secondary antibodies combined with HRP were incubated with the transfer membrane for 1 h. A strengthened chemiluminescence assay kit (Thermo Fisher Scientific, San Jose, CA, USA) was applied to detected Protein bands. Changes in protein expression were estimated using Image J.

Rabbit anti-p-JAK1 (Tyr1034/1035) antibody (CST#3331), rabbit anti-p-JAK2 (Tyr1007/1008) antibody (CST#3771), rabbit anti-p-JAK3 (Tyr980/981) antibody (CST#5031), rabbit anti-Caspase-1 antibody (CST#2225), rabbit anti-*β*-Actin mAb (CST#3700), rabbit anti-p-STAT1 (Tyr701) antibody (CST # 9167), and rabbit anti-Caspase-3 antibody (CST # 9662) were purchased from Cell Signaling Technology, Inc. (Shanghai, China).

### 2.6. Flow Cytometry Assay

NP cell apoptosis was worked out by the FITC-Annexin V/PI Apoptosis Detection kit (BD Pharmingen, San Diego, CA, USA) as stated by the instructions offered by the manufacturer. All cells were gathered after being transfected for 48 h. Propidium iodide (10 *μ*l) and Annexin V-FITC (5 *μ*l) were used to stain cells. After that, we applied a flow cytometer (Becton & Dickson, San Jose, California) to analyze the cell apoptosis.

### 2.7. TUNEL Assay

NP cells were collected from each group. After being fixed with 4% paraformaldehyde for 1 hour, it was cultured with 0.1%TritonX-100 for 10 minutes and cleaned with PBS 3 times. Depending on the specifications, the cells were stained within a situ apoptosis detection kit (F.Hoffmann LaRoche Ltd., Basel, Switzerland) and 40 (DAPI) 6-diamino-2-phenylindole. The absorbent paper dried the liquid on the climbing film, sealed the film with the sealing solution containing an antifluorescence quenching agent, and then, observed and collected the image under the fluorescence microscope.

### 2.8. Statistical Analysis

Dates of the experiment were demonstrated in the form of ±SD and evaluated in GraphPad Prism 8 (Graph Pad Software, La Jolla, CA, USA). Student's *t*-test or one-way ANOVA was used to estimate the difference. If the *P* value < 0.05, the statistic will be considered noteworthy.

## 3. Results

### 3.1. MiR-223 Upregulates the Expression of JAK2/STAT1 in NP Cells

**Figure 1:**
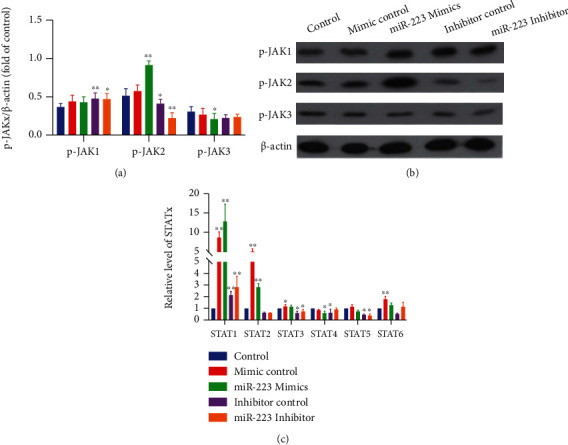
MiR-223 upregulates the expression of JAK2/STAT1 in NP cells. (a) The ratio of p-JAK1/p -JAK2/p-JAK3 to the internal reference. (b) WB band of p-JAK1/p-JAK2/p-JAK3. (c) Real-time manifestation of STAT1-STAT6 mRNA. ^∗^*P* < 0.05 vs. control; ^∗∗^*P* < 0.01 vs. control.

For the purpose of determining the impact of miR-223 on cell survival, miR-223 inhibitors or mimics were transfected in NP cell. The quality of STAT1-STAT6 was made out by qRT-PCR. The expression of p-JAK1/p-JAK2/p-JAK3 was recognized by WB. Contrasted with the control group, miR-223 mimics increased JAK2 and STAT1 expression markedly. However, miR-223 inhibitor declined JAK2 expression observably compared to control (Figures 1(a) and 1(c)).

### 3.2. MiR-223 Promotes LPS-Induced Inflammatory Response through JAK2/STAT1 Pathway in NP Cells

**Figure 2:**
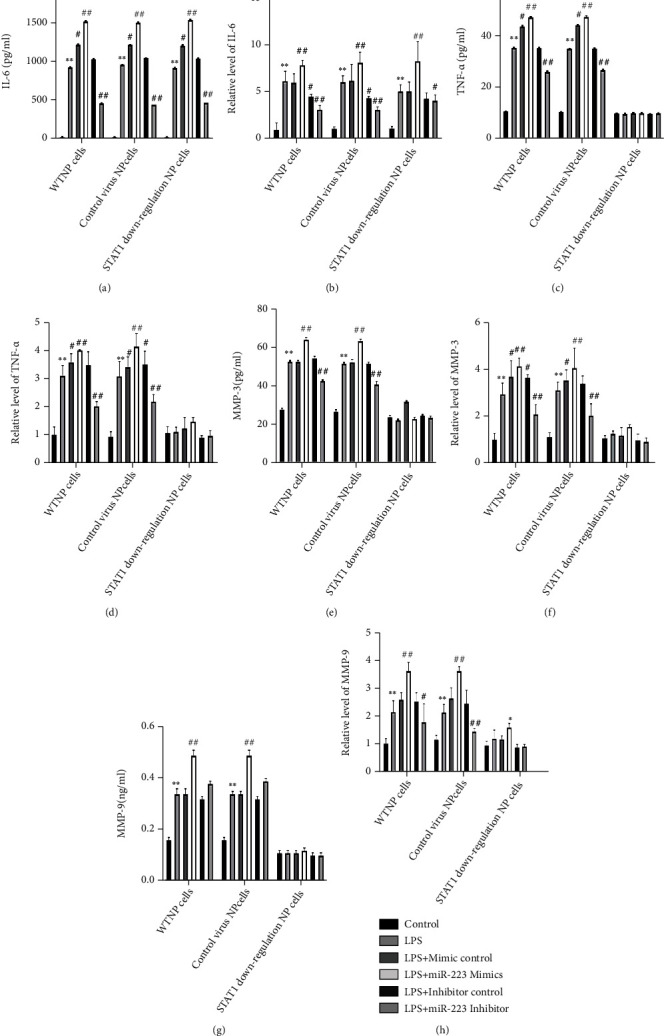
MiR-223 promotes LPS-induced inflammatory response through JAK2/STAT1 pathway in NP cells. The secretion levels of IL-6, TNF-*α*, and MMP-3, MMP-9 in WTNP cells, control virus NP cells, and STAT1 downregulation NP cells were measured by the ELISA method. The mRNA expression of IL-6, TNF-*α*, and MMP-3, MMP-9 in WTNP cells, control virus NP cells, and STAT1 downregulation NP cells was determined by the qPCR method. ^∗^*P* < 0.05 vs. control forth, *P* < 0.01 vs. control. ^#^*P* < 0.05 vs. miRmur 223 mimics control baffles, *P* < 0.01 vs. miRmur 223 mimics control *P* < 0.01 vs. miR-223 mimics control *P* < 0.01.

**Figure 3:**
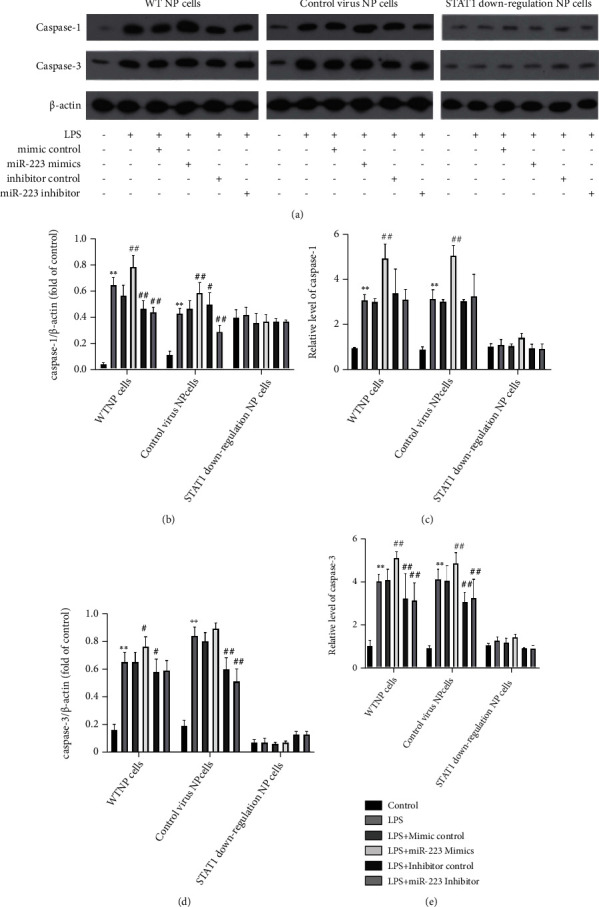
MiR-223 induces the manifestation of caspase-1 and caspase-3 in NP cells through JAK2/STAT1 pathway. WB bands of caspase-1 and caspase-3 in (a) WTNP cells, control virus NP cells, and STAT1 downregulation NP cells. The ratio of (b, d) caspase-1 and caspase-3 to internal reference. The real-time expression of (c, e) caspase-1 and caspase-3 mRNA. ^∗^*P* < 0.05 vs. control forth, *P* < 0.01 vs. control. ^#^*P* < 0.05 vs. miRmur 223 mimics control baffles, *P* < 0.01 vs. miRmur 223 mimics control *P* < 0.01 vs. miRmur 223 mimics control *P* < 0.01.

The shRNA lentivirus interference vector targeting STAT1 was constructed, and the NP cell line stably interfering with STAT1 gene was established. The expressions of TNF-*α*, IL-6, MMP-3, MMP-9, caspase-1, and caspase-3 in WTNP cells, control virus NP cells, and STAT1 downregulation NP cells were determined by ELISA, WB, and QPCR, respectively. In WTNP cells and control virus NP cells, miR-223 mimics significantly increased TNF-*α*, IL-6, MMP-3, MMP-9, caspase-1, and caspase-3 expression compared with the LPS group, and miR-223 inhibitor significantly decreased IL-6, TNF-*α*, MMP-3, and caspase-3 expression compared with the LPS group (Figures 2(a)–2(h) and 3(a)–3(e)). In STAT1 downregulation NP cells, miR-223 mimics could not increase the manifestation of TNF-*α*, MMP-3, MMP-9, caspase-1, and caspase-3, but significantly enhanced the expression of IL-6. Considering that miR-223 inhibitor cannot significantly inhibit the expression of caspase-1 in WTNP cells and control virus NP cells, while caspase-1 is mainly involved in the activation of interleukin precursors, the relationship between miR-223 and IL-6 and caspase-1 is worthy of further study.

### 3.3. MiR-223 Induces Apoptosis of NP Cells through JAK2/STAT1 Pathway

**Figure 4:**
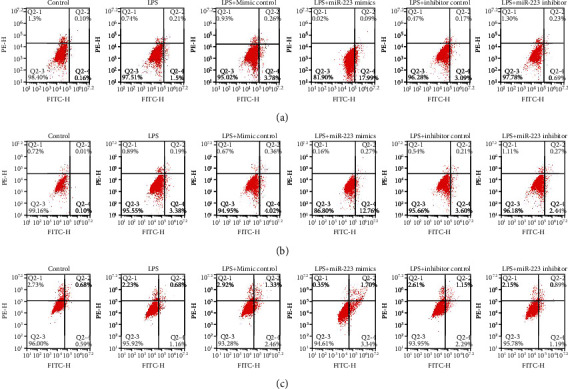
MiR-223 induces apoptosis of NP cells through JAK2/STAT1 pathway. Apoptosis of WTNP cells was detected by (a) flow cytometry. Apoptosis of control virus NP cells was detected by (b) flow cytometry. Apoptosis of STAT1 downregulation NP cells was detected by (c) flow cytometry.

**Figure 5:**
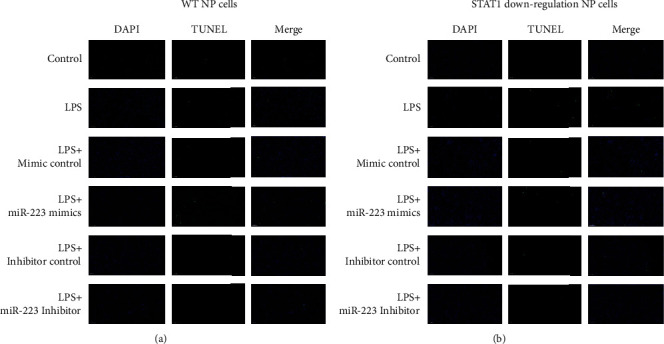
MiR-223 induces apoptosis of NP cells through JAK2/STAT1 pathway. (a) TUNEL staining showed apoptosis of WTNP cells. (b) TUNEL staining showed apoptosis of STAT1 downregulation cells.

**Figure 6:**
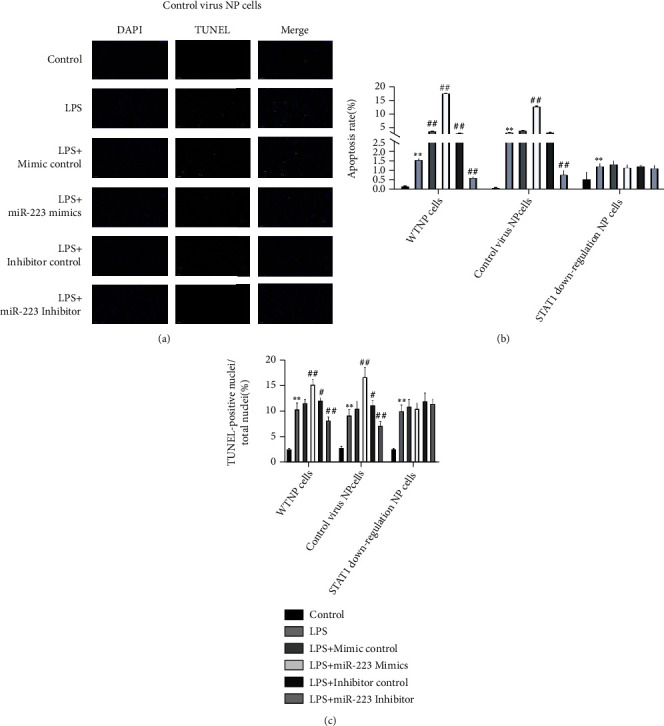
MiR-223 induces apoptosis of NP cells through JAK2/STAT1 pathway. (a) TUNEL staining showed apoptosis of control virus NP cells. (b, c) Flow cytometry and TUNEL staining were used to detect the apoptosis ratio of WTNP cells, control virus NP cells, and STAT1 downregulation NP cells. ^∗^*P* < 0.05 vs. control ability, *P* < 0.01 vs. control. ^#^*P* < 0.05 vs. miRmur 223 mimics controlitativs. *P* < 0.01 vs. miR-223 mimics control.

The apoptosis of WTNP cells, control virus NP cells, and STAT1 downregulation NP cells was detected by TUNEL staining and flow cytometry. After the three kinds of cells were treated with LPS, the proportion of the blank group was lower than that of apoptotic cells, indicating that LPS can induce the apoptosis of these three kinds of cells. The percentage of apoptotic cells in WTNP cells and control virus NP cells treated with LPS and MiR-223 mimics was superior to that in the LPS group, revealing that MiR-223 mimics can induce apoptosis. After (Figures 4(a), 4(b), 5(a), and 6(a) LPS and MiR-223 inhibitors coacting on two kinds of cells, the proportion of apoptotic cells is inferior to that in the LPS group, marking that MiR-223 inhibitor can promote the proliferation of these two kinds of cells. In STATx downregulation NP cells, the proportion of apoptosis in each group had no significant change compared with that in the LPS group (Figures 4(c), 5(b), 6(b), and 6(c)).

### 3.4. TS IIA Combined with AS IV Downregulates the Expression of miR-223 and JAK2/STAT1 in NP Cells

**Figure 7:**
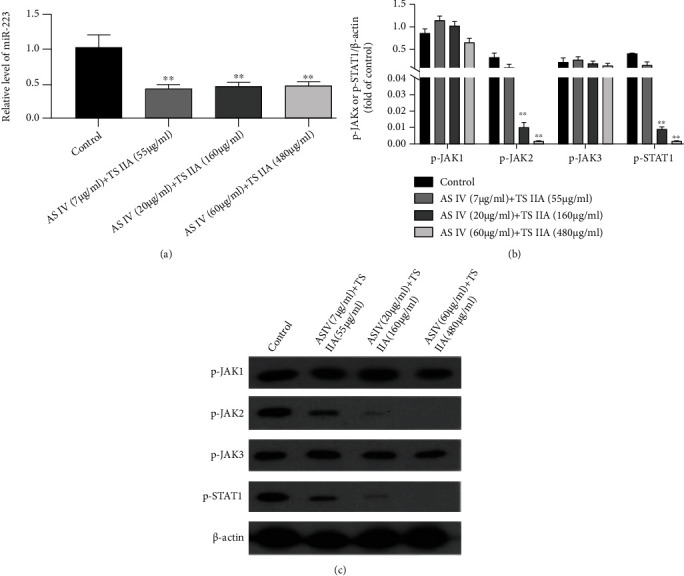
TS IIA combined with AS IV downregulated the manifestation of miR-223 and JAK2/STAT1 in NP cells. The effect of TS IIA combined with AS IV on the expression of miR-223 in NP cells at different doses of (a). The impact of TS IIA combined with AS IV on the manifestation of p-JAK1/p-JAK2/p-JAK3/p-STAT1 in NP cells at different doses of (b, c).

When NP cells were cotreated with TS IIA and AS IV, the representation of miR-223 in cells (Figure 7(a)) was inhibited at low concentrations. The results of WB detection revealed that the protein expression of p-JAK2 and p-STAT1 in NP cells was obviously decreased when TS IIA and AS IV were cotreated with AS IV, which indicated that TS IIA combined with AS IV could inhibit the expression of p-STAT1 and p-JAK2 in NP cells. Furthermore, the expression is displayed in a dose-dependent manner (Figures 7(b)and 7(c)). The protein manifestation of p-JAK1 and p-JAK3 in different concentrations of TS IIA and AS IV groups had no significant change compared with the blank group.

## 4. Discussion

The normal intervertebral disc consists of large numbers of extracellular matrix and a small number of cells, which account for only 1% of the intervertebral disc [21, 22]. The nucleus pulposus is decisive for the overall condition of the intervertebral disc, so current research on intervertebral disc degeneration is often focused on the nucleus pulposus. It is believed that IDD is related to the expression of inflammatory factors in cell matrix. Inflammatory cytokines and proteolytic enzymes can not only regulate the expression of proteins such as VEGF, FGF, and TGF and regulate microangiogenesis but also control the process of IDD. The main pathological changes of IDD were apoptosis of nucleus pulposus cells, inflammatory reaction, and excessive degeneration of ECM [23]. The decrease in the number of NP cells is an important factor leading to intervertebral disc degeneration [24, 25]. Although few pieces of research have summarized the detailed factors of miRNA in IDD, it has been proved that some miRNA can regulate nucleus pulposus cells, and a variety of miRNA is included in the apoptosis of NP cells [26–30]. The inflammatory factor is one of the important factors leading to the decrease of the number of nucleus pulposus cells. Intervertebral disc degeneration is related to varieties of proinflammatory cytokines IL-1 *β*, IL-6, and TNF-*α* and their metabolites [8, 31, 32]. However, in addition to protein regulators, miRNA is also a significant factor of inflammation, which mediates the signals of the occurrence and termination of inflammation and promotes or inhibits inflammation in different environments. For example, when miR155 decreased, the plentiful inflammatory intervenors such as IL-6 and TNF-*α* in mouse hepatocytes increased significantly. In this study, miR-223 mimics or miR-223 inhibitor was applied to NP cells.

It is known that the overexpression of inflammatory factors makes JAK phosphorylated and downstream STAT phosphorylated. P-STAT has transcriptional activation activity, which binds to the corresponding site, and then induces further activation of this pathway and promotes inflammation and apoptosis. JAK has four elements (JAK1, JAK2, JAK3, and TYK2). JAK3 exists only in the bone marrow and lymphatic system, and the remaining three kinds are widely distributed in cells and tissues of the body [33]. STAT, which is called a signal transducer and activator of transcription, belongs to cytoplasmic protein because it contains SH2 and SH3, which can bind to a specific peptide containing phosphorylated tyrosine through the DNA of the regulatory region of the target gene. STAT has seven kinds of family members (STAT1~4, STAT5a, STAT5b, and STAT6). STAT3 and STAT5 are most closely related to the human blood system. STAT contains SH2 and SH3 regions in structure, and SH2 and SH3 are between 600-700 and 500-600 amino acids, respectively. The sequence of SH2 is highly conserved, and SH3 is worse than SH2. Functionally, SH2 is related to the activation of STATS, while SH3 can bind to the sequence containing proline. However, in the signal transduction process of the JAK-STAT pathway, although one cytokine can activate a variety of JAK kinases, STAT can only be selected specifically [34]. For example, IL-4 activating STAT6 does not affect STAT3; IL-6 can activate STAT3 but does not affect STAT6. In this study, the upregulation of miR223 selectively promoted the demonstration of JAK2/STAT1 in NP cells, while the downregulation of miR-223 was on the contrary. Further studies showed that miR-223 mimics could not induce the expression of inflammatory and apoptosis-related factors such as TNF- *α*, Caspase-1, and Caspase-3 when reducing the expression of STAT1 in NP cells. Matrix metalloproteinases (MMP) are a group of Zn2+-dependent proteolytic enzymes, which are mainly involved in ECM metabolism, which are related to the remodelling of normal tissue and ECM degeneration under pathological conditions. MMP can act on a variety of components of intervertebral disc ECM, resulting in intervertebral disc degeneration [30]. The consequences of this research confirmed that the effect of miR-223 on the demonstration of MMP3 depended on the existence of STAT1, indicating that miR-223 mediated apoptosis and inflammation of NP cells by targeting JAK2/STAT1 and then promoted the degradation of intervertebral disc ECM and the occurrence of IDD.

The research group excavated the clinical data and found that Radix Astragali-Salvia miltiorrhiza is a commonly used compatibility in the treatment of traditional Chinese medicine [35–37]. In addition, Radix Astragali-Salvia miltiorrhiza has a therapeutic effect on lumbar disc herniation, cardio-cerebrovascular diseases, and so on. As the main components, tanshinone and astragaloside IV are expected to inhibit disc degeneration. In spite of there are various proportions of Salvia miltiorrhiza and Radix Astragali in traditional Chinese medicine, the best concentration of therapeutic effect has not been found. Based on this, we made a reasonable proportion of drugs with different concentrations to study the therapeutic effects of TSIIA and ASIV. This study provides a theoretical basis for the stage of pharmacological research to clinical application.

The inflammatory response of cells with different functions to JAK/STAT is different. Based on this study, we can speculate the following two points: (1) JAK/STAT pathway is involved in the inflammatory role of bone and joint diseases; (2) JAK2/STAT1 is negatively correlated with nucleus pulposus cell degeneration. This study has the following limitations: (1) Although the relationship between miR-223 and STAT1 was discussed in the experiment, the direct regulation experiment was not carried out to verify it. (2) The effects of tanshinone and astragaloside on the inflammatory reaction and extracellular matrix formation of nucleus pulposus cells were not tested. Based on the results of this study, we plan to continue to carry out follow-up experiments on the relationship between TSIIA, ASIV, and apoptosis and inflammation of NP cells. In addition, we will also bring animal experiments related to the effects of TSIIA and ASIV on the extracellular matrix into force.

Generally speaking, our study confirmed that miR-223 mediates apoptosis and inflammation of NP cells by targeting JAK2/STAT1 in vitro. TS IIA combined with AS IV inhibits the expression of miR-223 and regulates JAK2/STAT1 pathway. Our experimental results support the potential of miR-223 as a bioindicator for IDD therapy. Since many JAK blockers have been used in clinics [38, 39], this study also provides a targeted basis for new small molecular JAK blockers.

## Data Availability

The data used to support the findings of this study are available from the corresponding author upon request.
